# A Photochromic Sensor Microchip for High-performance Multiplex Metal Ions Detection

**DOI:** 10.1038/srep09724

**Published:** 2015-04-08

**Authors:** Yu Huang, Fengyu Li, Changqing Ye, Meng Qin, Wei Ran, Yanlin Song

**Affiliations:** 1 Key Laboratory of Green Printing, Institute of Chemistry, Chinese Academy of Sciences; 2 University of the Chinese Academy of Sciences

## Abstract

Current multi-analytes chips are limited with requiring numbers of sensors, complex synthesis and compounds screen. It is expected to develop new principles and techniques to achieve high-performance multi-analytes testing with facile sensors. Here, we investigated the correlative multi-states properties of a photochromic sensor (spirooxazine), which is capable of a selective and cross-reactive sensor array for discriminated multi-analytes (11 metal ions) detection by just one sensing compound. The multi-testing sensor array performed in dark, ultraviolet or visual stimulation, corresponding to different molecular states of spirooxazine metal ions coordination. The facile photochromic microchip contributes a multi-states array sensing method, and will open new opportunities for the development of advanced discriminant analysis for complex analytes.

There is an increasing need of convenient, rapid, sensitive and high-throughput sensors for target multi-analytes identification in the environmental monitoring^1^,^2^,^3^,^4^, aliment safety^5^,^6^, clinical diagnoses^7^,^8^,^9^,^10^,^11^,^12^,^13^,^14^, and so on^15^,^16^. Sensor arrays are a verified valid detection method in the area of multi-analytes testing and high-throughput analysis^17^,^18^,^19^,^20^. Currently, a graphene-based ensemble aptamers were exploited to recognize molecular or cellular targets discriminatively^10^. Cui *et al.* developed a sensor array assembled from polyionic liquid inverse opaline microspheres to test five anions^16^. Generally, the sensor array performed the correlative differential analyzing needs large numbers of serial compounds or complicated compound with several recognition groups as sensor, which involves complicated chemical synthesizing and valid compounds screening. Examples include adopting six various 8- hydroxy-quinoline derivants to realize metal ions detection^21^, designing sensor combining four hydrophobic recognition groups to take molecular diagnostics^22^, seven sensors utilized a common calix[4]pyrrole receptor to detect 14 carboxylates^23^. Although the serial sensors own high sensitivity, the high production cost and challenges regarding valid compounds screen restrict their practical applications. These facts call for a reliable sensor to alleviate these concerns for general high-performance multi-analytes detection. Here, we adopt a photochromic material, spirooxazine, to multi-testing sensor array, and utilize the multi-states of the sensing array under stimulations acting the selective and cross-reactive sensing (Fig. 1). This photochromic sensor microchip can actualize eleven various metal ions detection with only one facile sensor, which offers a new strategy to design and develop high-performance multi-testing sensor array.

Spiropyran and spirooxazine (SP), which respond to different external stimulations, such as light, proton and metal ions to undergo reversible structural interconversion accompanied the change in photophysical properties, are of interest in the emerging area of logic gates and communication networks^24^,^25^,^26^, molecular switches and unit^27^,^28^, data storage^29^, metal ions detection and other fields^30^,^31^,^32^,^33^,^34^,^35^,^36^,^37^. With different light stimulations, spiropyran and spirooxazine interconvert among the closed form to merocyanine form (MC). The merocyanine spiropyran/spirooxazine can combine with proton or metal cations to form metallic merocyanine (MMC), as shown in Fig. 2^24^,^25^,^26^,^30^,^31^,^32^,^33^,^34^,^35^,^36^,^37^. Performing the reversible reactions between closed, MC and MMC forms of spiropyran/spirooxazine, different ions strength and light stimulations have the differential inductive effect. It gives abundant chemical information in forms of interconvert processing, which can be adopted to high-throughput and complex analysis. Since fluorescent detection owns the significant advantages of high-sensitive, quick-response and non-contacting, we choose a commercial spirooxazine: 1, 3, 3-trimethylindolinonaphthospirooxazine to build a photochromic sensor microchip for high-performance multiplex metal ions detection.

## Results

**Figure 3:**
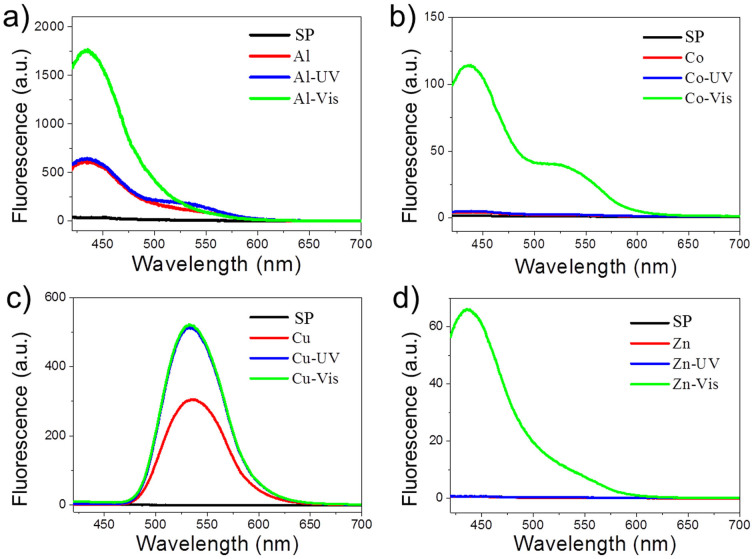
The fluorescence of spirooxazine response to various metal ions with different light irradiations. The typical fluorescent spectra of spirooxazine in ethanol (1.0 mM) response with Al^3+^, Co^2+^, Cu^2+^, Zn^2+^ (1.0 mM) in dark, after UV-light or Vis-light irradiation, respectively.

Fig. 3 displays the typical fluorescence spectra of spirooxazine in ethanol (1.0 mM) response with metal ions in different light-stimulations. Spirooxazine combining with Al^3+^ has obvious fluorescence increasement, ultraviolet (UV) light irradiation grows the 533 nm fluorescence and visible (Vis) light irradiation drastically increases the fluorescent peak of 435 nm. In the case of spirooxazine response with Co^2+^, Vis light stimulation increases the peaks of 435 and 533 nm of the fluorescent spectra. Cu^2+^ has the strong coordinating ability with spirooxazine/spiropyran and results in the closed form ring-open. The spirooxazine-Cu^2+^ coordination generates a medium fluorescence. UV and Vis irradiation further enhance the fluorescence at 533 nm. Spirooxazine-Zn^2+^ coordination dose not contribute the obvious fluorescence when prime stain and UV irradiation. The electron transfer from Zn^2+^ to organic ligand under Vis irradiation contributes a weak fluorescence emission. The change of fluorescence spectra of spirooxazine combined with Al^3+^, Co^2+^, Cu^2+^, Zn^2+^ under dark, UV and Vis irradiation are focus at the wavelength of 435 nm and 533 nm because of the light stimulations induce the merocyanine of the spirooxazine-metallic coordinations partly convert to open or closed form. Furthermore, when this sensing system was adopted in cross-reactive array sensing, fluorescent signals were collected in the same condition at the same time, which will avoid error from excitation light source glint, detector fatigue. Accurate chemical difference was present in this sensing array system. The phenomena that spirooxazine-metallic coordinations reveal various fluorescent spectra in different light stimulations demonstrate the sensing molecular spirooxazine can provide multiple fluorescent signals, which will contribute to the photochromic sensor microchip selective and cross-reactive testing.

**Figure 4:**
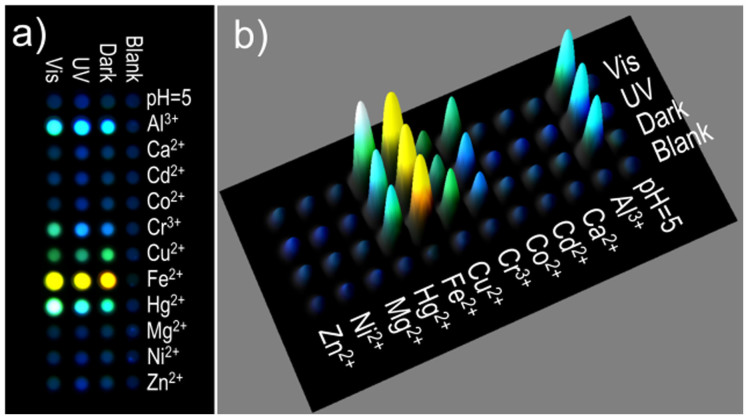
(a) Fluorescent image and (b) 3D representation of the integrated fluorescence intensity of the photochromic SP microchip constitute from rows from each light irradiations process of the photochromic microchip. The Fluorescent image of photochromic microchip spotted by metal ions (1.0 mM in water, 200 nL), which was recorded in 6 different channels. The colours were generated by superimposing of the equally weighed images corresponding to RGB channels.

Based on the fluorescent enhancement or shift of different spirooxazine-metallic coordination states, we designed a microchip adopting spirooxazine as the only one sensor, analysing the fluorescent signals to realize multiple metal ions detection. To generate the cross-reactive SP microchip, we carried out 1.0 mM 1, 3, 3-trimethylindolinonaphthospirooxazine and thermoplastic polyurethane Tecoflex® (0.5% PU in Ethanol) solution (200 nL) pipetted onto each pixel of the microchip. Here we used PU as the interface medium for the sensing in each pixel. PU is a block polymer with terephthalic acid units and ethylene glycol units, which is solvable in ethanol and swellable in water. On this microchip, AlCl_3_, CaCl_2_, CdCl_2_, CoCl_2_, CrCl_3_, CuCl_2_, FeCl_2_, HgCl_2_, MgCl_2_, NiCl_2_ and ZnCl_2_ (1.0 mM in water, 200 nL, pH = 5) and pH = 5 control aqueous solutions (HCl-NaCl) were spotted on corresponding pixels in 12 rows respectively. The fluorescence responses of the photochromic spirooxazine microchip to the presence of 11 metal ions (Al^3+^, Ca^2+^, Cd^2+^, Co^2+^, Cr^3+^, Cu^2+^, Fe^2+^, Hg^2+^, Mg^2+^, Ni^2+^ and Zn^2+^) were recorded in 6 channels (CH 1: 450 nm, CH 2: 480 nm, CH 3: 505 nm, CH 4: 535 nm, CH 5: 570 nm and CH 6: 605 nm, with UV 365 nm excitation). The colours were generated by superimposing of the equally weighed images corresponding to RGB channels. Dark, UV and Vis stimulation resulted in the spirooxazine microchip displaying various fluorescent signals. For better comparing the fluorescent difference of dark, UV and Vis process, rows from each light irradiation steps were selected to constitute Fig. 4. The fluorescent image in Fig. 4b displays obvious different fluorescence intensity and shift for each row, which will contribute more information for high-performance multi-testing.

**Figure 5:**
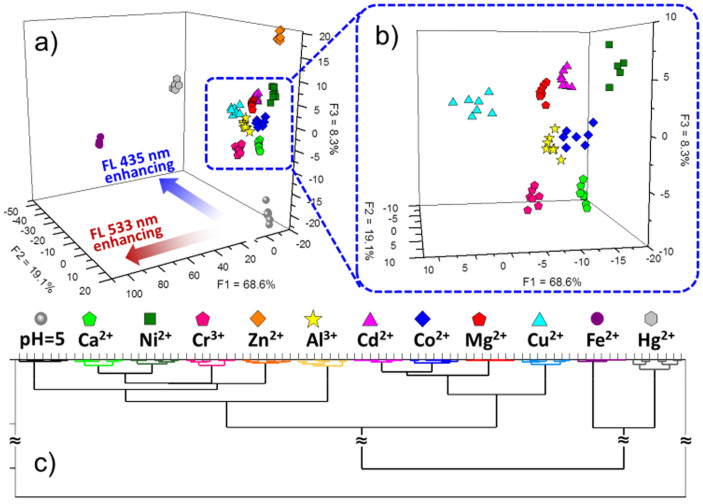
Fluorescent discriminant analysis of 11 metal ions on SP multi-states microchip and rational analysis. (a, b) Graph of LDA result shows a clear clustering of the 11 metal ions analytes and its corresponding magnified image. LDA reflects analytes specific fluorescent enhancement at 435 nm or 533 nm due to various metal electropositivity. The gathering of the clusters demonstrates the good repeatability of the SP multi-states microchip for each metal ion response. (c) HCA gives the similarity clustering of the analytes based on the fluorescent variation trend of the spirooxazine-metallic coordination in three states.

The discriminatory capability of the photochromic SP microchip can be performed using multivariate analysis. Linear discriminant analysis (LDA) was used to evaluate the similarities between the data corresponding to the same cluster by introducing the group classification^38^,^39^,^40^,^41^,^42^. LDA provides a graphic representation useful to gain an insight into the clustering of the response data, and to calculate classification accuracy. Fig. 5a, b represent the LDA score plot of the first three dispersion factors (**F1–F3**) describing of the total variance, which displays the clear clustering of the data. Since LDA trains the data to describe the best-fit parameters to separate different clusters, the distance of the cluster in spatial distribution reveals differential fluorescent signal of the metal ions. Obviously, the LDA score plot shows clustering for all 12 samples (7 trials each, 1.0 mM). It gives the 100% correct clustering of the eleven metal ion samples and one control sample water pH = 5 (Table S1, Supporting Information [SI]). The clusters were separated in the space of differential fluorescence peak (435 or 533 nm) distribution orientation. Furthermore, we also adopted hierarchical clustering analysis (HCA) to carrier out multivariate analysis. HCA performs dimensionality reduction analysis to investigate the similarity clustering of the analytes. In this work, we defined the cluster by Ward's (minimum variance) method^43^, which takes into consideration the minimum amount of variance between the samples. The HCA result shows a dendrogram of euclidean distance between 84 samples with ward linkage in Fig. 5c and Fig. S1. HCA graphical output displays three major groups, which indicates fluorescent variation trend of the different states of spirooxazine-metallic coordination. Overall evaluating the LDA and HCA, the results reflect the original information of sensing, which indicates subtle and accurate details for the photochromic SP microchip sensing various metal ions. The spirooxazine-metallic responses focus on the two major peaks of 435 and 533 nm on fluorescence spectra. The different fluorescence intensity ratios between 435 and 533 nm reveal various classification contributions of photochromic SP microchip in different light stimulations.

## Discussion

To evaluate the classification efficiency of the photochromic SP microchip with various states, we explored the LDA of SP array in dark, UV or Vis stimulation state separately (Table S2–S4). None of the single state of SP array can result in a completely correct classification individually. The LDA results showing in Table S5 displays the near and confused spatial distribution of the clusters in each single state of SP array, which reveals the small differential of fluorescent signals of the metal ions in these analysis systems. In details, the SP sensing array in dark state can discriminate Al^3+^, Cr^3+^, Cu^2+^, Fe^2+^, Hg^2+^, Mg^2+^, Ni^2+^ and Zn^2+^, and cannot correctly analysis Ca^2+^, Cd^2+^, Co^2+^ and pH = 5. UV state does not improve the general accuracy than dark state, but it increases the accurate rate for Ca^2+^ identification and help to distinguish misclassed Co^2+^ from control (pH = 5). In the case of Vis state, the microchip generates the clear identification of Al^3+^, Cd^2+^, Co^2+^, Cr^3+^, Cu^2+^, Fe^2+^, Hg^2+^, Mg^2+^, Zn^2+^ and pH = 5, only has deficiency at Ca^2+^ and Ni^2+^. The three states are highly complementary in distinguishing metal ions. It guarantees the integrated SP multi-states array to perform the 100% correct clustering of the eleven metal ion samples with one control sample.

The key point of sensor array successful in multi-targets analysis is to provide enough differential and correlative signals. However, most single sensing molecule lacks enough differential signals to correctly cluster of the multi-analytes. Previous researches usually focused on developing serial sensors to get correlatively differential messages^7^,^8^,^9^,^10^,^11^,^12^,^13^,^14^,^15^,^16^,^17^,^18^,^19^,^20^,^21^. The strategies usually are preparing sensor array with serial sensor compounds to plus and fold the correlative response difference, or synthesizing special and complex sensor compound to generate obviously discriminated response. Here, the single photochromic sensing compound gives a strong correlativity between the configurations interconverting under different light stimulations. The differential states under dark, UV and Vis stimulation generate differential and correlative signals, which perform as three serial sensors adopted in this sensing array and contribute to perform a correct discriminant analysis. By the well-known Kolmogorov's law of the iterated logarithm^44^, the relation of multi-targets analysis successful probability (***P***) and participant observation states (***n_i_***) can be deduced as (For the detailed deduction process, please see Supporting Information):
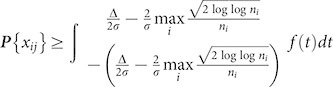


There are many factors contribute to the probability of success of multi-targets analysis, here we focus on the influence of *n_i_*. The *x_ij_* presents the classification of success. The mathematical deduction suggests the universality of the strategy applied in multi-targets analysis. It can be simply described as a monotonically increasing function. That is meaning the calculated probability of success obviously increases when the observation's number increases, which is well in agreement with experiment results. The multi-states analysis method guarantees the generated signals cross-reactive and differential, which realizes high-performed multi-analytes identification on a single sensing compound array, and avoids the complex process of serial synthesis and valid compound screening.

**Figure 6:**
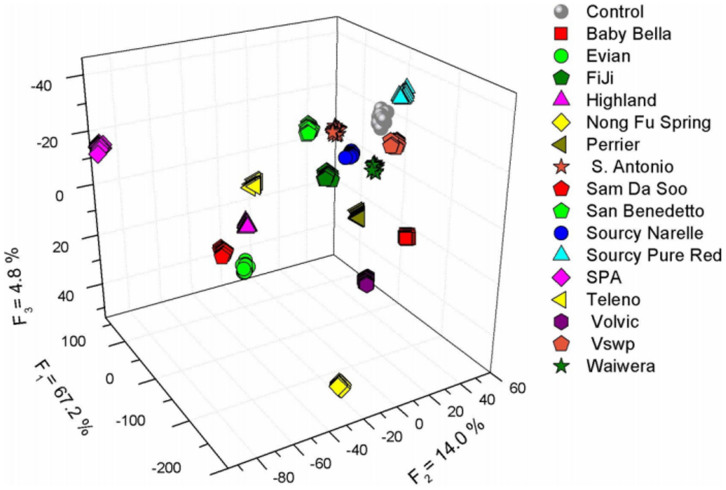
LDA score plot corresponding to the response of the SP microchip to 16 kinds of natural mineral water comes from various countries. The data set contains 16 brands and 1 control, 15 trials each. LDA shows 100% correct classification for all water brands.

Encouraged by the results above, we explored the utility of the spirooxazine multi-states sensor array by exploring a potential application: identification of mineral and purified water (Milli-Q water) based on their cation content as shown in Fig. 6. Table 1 lists Ca^2+^ and Mg^2+^ contents for all of the mineral water brands. The pH levels of most brands are in the range of 6–7.8 (except the Sourcy pure red with pH = 4.7), where the sensor array presents a rather flat response. The list is clear that all 16 commercial potable water samples contain different concentrations of cations. LDA analysis shows a complete and clear 100% correct classification for all 255 trials (Figure S6). The study demonstrates that the photochromic SP microchip detection of metal ions can be generated into metal ions mixtures. Furthermore, we also processed the photochromic SP microchip for detecting metal ions in human serum (protein removed), and the LDA result displays 100% classification accuracy (Fig. S2, Table S7). The result suggests its potential applications in complex physiological environment.

In conclusion, utilizing interconversion of photochromic materials, we developed a high-performance multi-analytes sensor array with a single compound. Significantly, this sensor array is capable of identification of 11 metal ions, based on multi-states of spirooxazine in UV and Vis irradiations. The successful discriminating of various natural mineral water samples proves the photochromic SP microchip practical application in mixed and complicated analysis. It contributes a new idea of stimulated (photo-, electro-, magnet-, thermo-, chemo-, *etc*) conversion sensor array for multi-testing, which will have implicative applications in chemical, biological or medicinal samples testing. The future complex sample detections for pollution monitoring, aliment safety and clinical diagnoses will be facile, efficiency and low-cost with the high-performance sensing and analysis method.

## Methods

### Fluorescence testing

A UV LED lamp with the wavelength of 245 nm was used as the UV illumination source. Fluorescence measurements were performed in a Hitachi F-4500 Fluorescence Spectrophotometer. The absorption spectra were measured with a fiber optic UV-Vis-IR spectrometer (Ocean Optic HR 4000 CG).

### Microchip sensing

1.0 mM 1, 3, 3-trimethylindolino-naphthospirooxazine and thermoplastic polyurethane Tecoflex® (0.5% PU in Ethanol) solution (200 nL) was pipetted onto each pixel of the microchip. The microchip was performed to the multi-analytes testing (11 metal ion plus the pH = 5 control solution). On the microchip, AlCl_3_, CaCl_2_, CdCl_2_, CoCl_2_, CrCl_3_, CuCl_2_, FeCl_2_, HgCl_2_, MgCl_2_, NiCl_2_ and ZnCl_2_ (1.0 mM in water, 200 nL, pH = 5) and pH = 5 control aqueous solutions (HCl-NaCl) were spotted on corresponding pixels respectively. The fluorescence responses of the microchip to different metal ions were recorded by fluorescence scanner (ChampChemi Professional+) in six channels (CH1: 450 nm, CH2: 480 nm, CH3: 505 nm, CH4: 535 nm, CH5: 570 nm and CH6: 605 nm, with 365 nm UV light excitation). The data processing consisted on the integration of the fluorescence intensity per pixel of the microchip before and after the solutions of metal ions spotted.

### Data analysis

The statistical multivariate methods, LDA and HCA^38^,^39^,^40^,^41^,^42^ are routinely used to interpret and evaluate the responses from cross-reactive sensor arrays, providing a graphical output useful to gain an insight into the clustering of the response data, and calculate classification accuracy. The metal ion spots were formed on microchip and record in six channels by fluorescence scanner. The LDA was carried out using SYSTAT® v12.02.00 and the HCA was carried out using Minitab® v16.1.1.0.

## Author Contributions

Y.H., F.L. and Y.S. conceived and designed the experiments. Y.H., F.L. and M.Q. performed the experiments. Y.H., F.L., C.Y. and W.R. analysed the data and wrote the manuscript. All authors reviewed the manuscript.

## Supplementary Material

Supplementary Information Supplementary Information

## Figures and Tables

**Figure 1:**
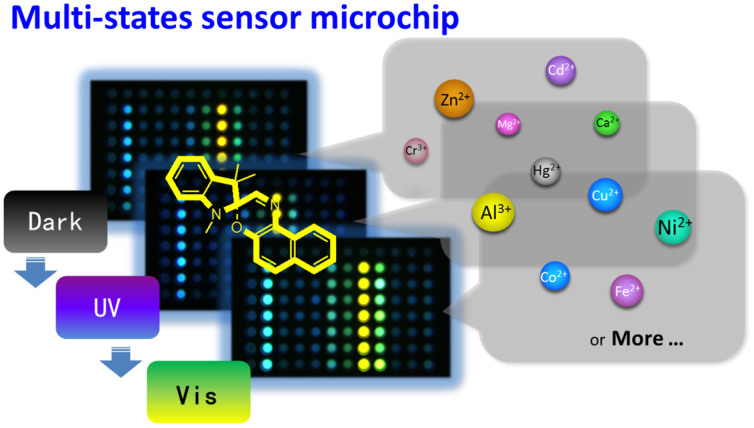
The schematic illustration of the photochromic multi-states sensing for multi-analytes metal ions detection with one spirooxazine sensor. Three states (dark, UV irradiation and Vis irradiation) of spirooxazine-metallic coordination can be obtained by spirooxazine response with metal ions in different irradiation conditions.

**Figure 2:**
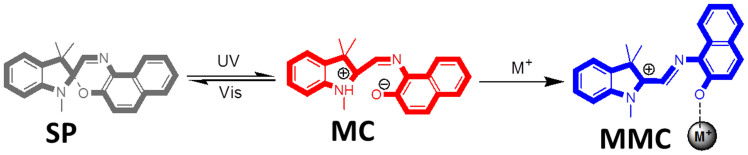
The interconverts of spirooxazine among the closed form, merocyanine (MC) form and metallic merocyanine (MMC) form.

**Table 1:** Metal ion content for different brands of mineral water samples. The samples contain different kinds and concentrations of metal ions

Metal Ion Content (mg/L)							
Mineral Water	Ca	Mg	pH	Mineral Water	Ca	Mg	pH
Baby Bella	53.3	14.9	-	Evian	80	26	7.2
FIJI	17.5	-	7.7	Highland	40	10.1	7.8
Nong Fu Spring	40	5	7.3	Perrier	160	4.2	-
S. Antonio	32.4	5.3	7.9	Sam Da Soo	2.9	1.9	7.7
San Benedetto	50.3	30.8	7.52	Sourcy Naturelle	54	4.5	7.5
Sourcy Pure Red	54	4.5	4.7	SPA	4.5	1.3	6
Teleno	6	1.2	-	Volvic	11.5	8.0	7
Vswp	45	51	-	Waiwera	12	2.6	7.6

## References

[b1] Lim S. H. *et al.* An optoelectronic nose for detection of toxic gases. Nat. Chem. 1, 562–567 (2009). 20160982 10.1038/nchem.360 PMC2761044

[b2] Perry R. H. *et al.* Detecting reaction intermediates in liquids on the millisecond time scale using desorption electrospray ionization. Angew. Chem. Int. Ed. 50, 250–254 (2011). 10.1002/anie.201004861 21110361

[b3] Yang Y. J. *et al.* A highly selective low-background fluorescent imaging agent for nitric oxide. J. Am. Chem. Soc. 132, 13114–13116 (2010). 20672823 10.1021/ja1040013

[b4] Xie Z. *et al.* An optical nose chip based on mesoporous colloidal photonic crystal beads. Adv. Mater. 26, 2413–2418 (2013). 24375812 10.1002/adma.201304775

[b5] Lavigne J. J. & Anslyn E. V. Sensing a paradigm shift in the field of molecular recognition: from selective to differential receptors. Angew. Chem. Int. Ed. 40, 3119–3130 (2001). 10.1002/1521-3773(20010903)40:17<3118::AID-ANIE3118>3.0.CO;2-Y 29712042

[b6] Huang Y., Li F. Y., Qin M., Jiang L. & Song Y. L. A multi-stopband photonic-crystal microchip for high-performance metal-ion recognition based on fluorescent detection. Angew. Chem. Int. Ed. 125, 7437–7440 (2013). 10.1002/anie.201302311 23754546

[b7] Buryak A. & Severin K. A chemosensor array for the colorimetric identification of 20 natural amino acids. J. Am. Chem. Soc. 127, 3700–3701 (2005). 15771496 10.1021/ja042363v

[b8] Zaubitzer F. Buryak A. & Severin K. Cp*Rh-based indicator-displacement assays for the identification of amino sugars and aminoglycosides. Chem. Eur. J. 12, 3928–3934 (2006). 16521137 10.1002/chem.200501410

[b9] Liu W. *et al.* Photonic crystal encoded microcarriers for biomaterial evaluation. Small 10, 88–93 (2014). 23861358 10.1002/smll.201301253

[b10] Pei H. *et al.* Designed diblock oligonucleotide of DNA-gold nanoparticle nanoconjugates. J. Am. Chem. Soc. 134, 13843–13849 (2012). 22799460 10.1021/ja304118z

[b11] Shao N. *et al.* A spiropyran-based ensemble for visual recognition and quantification of cysteine and homocysteine at physiological levels. Angew. Chem. Int. Ed. 45, 4944–4948 (2006). 10.1002/anie.200600112 16810651

[b12] Adams M. & Anslyn E. V. J. Am. Chem. Soc. 131, 17068–17069 (2009). 19904949 10.1021/ja908319m

[b13] De M. *et al.* Sensing of proteins in human serum using nanoparticle-green fluorescent protein conjugates. Nat. Chem. 1, 461–465 (2009). 20161380 10.1038/nchem.334 PMC2782604

[b14] Miranda O. *et al.* Enzyme-amplified array sensing of proteins in solution and in biofluids. J. Am. Chem. Soc. 132, 5285–5289 (2010). 20329726 10.1021/ja1006756 PMC2855490

[b15] Grinthal A. & Aizenberg J. Adaptive all the way down: building responsive materials from hierarchies of chemomechanical feedback. Chem. Soc. Rev. 42, 7072–7085 (2013). 23624804 10.1039/c3cs60045a

[b16] Cui J. *et al.* Inverse opal sphere based on polyionic liguids as functional microspheres with tunable optical properties and molecular recognition capabilities. Angew. Chem. Int. Ed. 53, 3844–3848 (2014). 10.1002/anie.201308959 24596228

[b17] Carey J. *et al.* Rapid identification of bacteria with a disposable colorimetric sensing array. J. Am. Chem. Soc. 133, 7571–7576 (2011). 21524080 10.1021/ja201634d PMC3097425

[b18] Bajaj A. *et al.* Array based sensing of normal, cancerous and metastatic cells using conjugated fluorescent polymers. J. Am. Chem. Soc. 132, 1018–1022 (2010). 20039629 10.1021/ja9061272 PMC2810251

[b19] Greene N. & Shimizu K. D. Colorimetric molecularly imprinted polymer sensor array using dye displacement. J. Am. Chem. Soc. 127, 5695–5700 (2005). 15826210 10.1021/ja0468022

[b20] Rochat S. *et al.* Cross-reactive sensor arrays for the detection of peptides in aqueous solution by fluorescence spectroscopy. Chem. Eur. J. 16, 104–113 (2010). 19938007 10.1002/chem.200902202

[b21] Palacios M. A. *et al.* Rational design of a minimal size sensor array for metal ion detection. J. Am. Chem. Soc. 130, 10307–10314 (2008). 18616249 10.1021/ja802377k

[b22] Rout B., Unger L., Armony G., Iron M. A. & Margulies D. Medication detection by a combinatorial fluorescent molecular sensor. Angew. Chem. Int. Ed. 51, 12477–12481 (2012). 10.1002/anie.201206374 23065749

[b23] Liu Y. *et al.* Sensing of carboxylate drugs in urine by a supramolecular sensor array. J. Am. Chem. Soc. 135, 7705–7712 (2013). 23656505 10.1021/ja4015748

[b24] Kubinyi M. *et al.* Metal complexes of the merocyanine form of nitrobenzospyran: structure optical spectra, stability. Journal of Molecular Structure 1000, 77–84 (2011).

[b25] Guo X. F., Zhang D. Q. & Zhu D. B. Logic control of the fluorescence of a new dyad, spiropyran-perylene diimide-spiropyran, with light, ferric Ion, and proton: construction of a new three-input “AND” logic gate. Adv. Mater. 16, 125–130 (2004).

[b26] Kato S. *et al.* Homoconjugated push-pull and spiro systems: intramolecular charge-transfer interactions and third-order optical nonlinearities. Angew. Chem. Int. Ed. 49, 6207–6211 (2010). 10.1002/anie.201002236 20648502

[b27] Tian H. *et al.* A single photochromic molecular switch with four optical outputs probing four inputs. Adv. Mater. 15, 2104–2107 (2003).

[b28] Bahr L. *et al.* Photoswitched singlet energy transfer in a porphyrin-spiropyran dyad. J. Am. Chem. Soc. 123, 7124–7133 (2001). 11459493 10.1021/ja010058t

[b29] Yuan W. F. *et al.* A novel thermally stable spironaphthoxazine and its application in rewritable high density optical data storage. Adv. Mater. 17, 156–160 (2005).

[b30] Ren J. Q. & Tian H. Thermally stable merocyanine form of photochromic spiropyran with aluminum ion as a reversible photo-driven sensor in aqueous solution. Sensors 7, 3166–3178 (2007). 10.3390/s7123166 PMC3841888 28903287

[b31] Natali M. & Giordani S. Interaction studies between photochromic spirospyrans and transition metal cations: the curious case of copper. Org. Biomol. Chem. 10, 1162–1171 (2012). 22146800 10.1039/c1ob06375h

[b32] Zakharova M. *et al.* Quantitative investigations of cation complexation of photochromic 8-benzothiazole-substituted benzopyran: towards metal-ion sensors. Photochem. Photobiol. Sci. 9, 199–207 (2010). 20126795 10.1039/b9pp00112c

[b33] Fies K. *et al.* Spectroscopic analysis of metal ion binding in spiropyran containning copolymer thin films. Anal. Chem. 82, 3306–3314 (2010). 20345120 10.1021/ac1001004

[b34] Fries K. *et al.* Fabrication of spirophyran-containning thin film sensors used for the simultaneous identification of multiple metal ions. Langmuir 27, 12253–12260 (2011). 21877693 10.1021/la202344w

[b35] Nishikiori H. *et al.* Photochromic behavior of spironaphthoxazine in metal ion-containing solutions. Journal of Photochemistry and Photobiology A: Chemistry 222, 236–240 (2011).

[b36] Zakharova M. I. *et al.* Thermodynamic and kinetic analysis of metal ion complexation by photochromic spiropyrans. RUSSIAN CHEMICAL BULLETIN. 58, 1329–1337 (2009).

[b37] Zhang H. T. *et al.* Interface engineering of semiconductor/dielectric heterojunctions toward functional organic thin-film transistors. Nano Lett. 11, 4939–4946 (2011). 22011136 10.1021/nl2028798

[b38] Shabbir S. H., Regan C. J. & Anslyn E. V. A general protocol for creating high-throughput screening assays for reaction yield and enantiomeric excess applied to hydrobenzoin. Proc. Natl. Acad. Sci. U.S.A. 106, 10487–10492 (2009). 19332790 10.1073/pnas.0809530106 PMC2705572

[b39] Umali A. P. & Anslyn E. V. A general approach to differential sensing using synthetic molecular receptors. Curr. Opin. Chem. Biol. 14, 685–692 (2010). 20801075 10.1016/j.cbpa.2010.07.022 PMC2997874

[b40] Beebe K. R., Pell R. J. & Seasholtz M. B. In Chemometrics: a practical guide. (Wiley: New York, 1998).

[b41] Otto M. In Chemometrics: Statistics and computer application in analytical chemistry. (Wiley-VCH: New York, 1999).

[b42] Jambu M. In Exploratory and MultiVariate Data Analysis. (Academic Press, San Diego, 1991).

[b43] Ward J. H. Hierarchical grouping to optimize an objective function. J. Am. Stat. Assoc. 58, 236–244 (1963).

[b44] Kolmogoroff A. Über das Gesetz des iterierten Logarithmus. Mathematische Annalen 101, 126–135 (1929).

