# Supplementary material for: A Photochromic Sensor Microchip for High-performance Multiplex Metal Ions Detection
Source: Sci Rep. 2015 Apr 8;5:9724. doi: 10.1038/srep09724 (PMC4389811; doi:10.1038/srep09724)
Supplement: Supplementary Information [file srep09724-s1.doc]

**Supporting Information for**

**A Photochromic Sensor Microchip for High-performance Multiplex Metal Ions Detection**

*Yu Huang^1,2^, Fengyu Li^1^,*, Meng Qin^1,2^, Changqing Ye^1^, Wei Ran^1,2^, and Yanlin Song^1^,**

*^1^Beijing National Laboratory for Molecular Sciences (BNLMS)*

*Laboratory of New Materials, Key Laboratory of Organic Solids, Institute of Chemistry, Chinese Academy of Sciences, Zhongguancun North First Street 2,100190 Beijing, PR China.*

*^2^University of Chinese Academy of Sciences, No.19A Yuquan Road, Beijing 100049, China*

**E-mail:* [*forrest@iccas.ac.cn*](mailto:forrest@iccas.ac.cn) *&* [*ylsong@iccas.ac.cn*](mailto:ylsong@iccas.ac.cn)

**Contents:**

**General**………………………………………………………………………………2

**Experimental section**……………………………………..………………………..…2

**Data analysis**……………………………………………………………………….…3

Linear discriminant analysis (LDA)……………………………………………….…3

Hierarchical clustering analysis (HCA)………………………………………….…7

LDA analysis of 16 kinds of natural mineral water…………………………….…9

LDA analysis of 11 metal ions in human serum……………………………………10

**Mathematical deduction**……………………………………………………………11

**Reference**………………………………………………………………………….…13

**■ General**

Commercially available solvents and reagents were used as received from the chemical suppliers, including the sensing molecular 1, 3, 3-trimethylindolinonaphthospirooxazine (CAS Number: 27333-47-7). Fluorescence measurements were performed in an *Hitachi* F-4500 Fluorescence Spectrophotometer. Fluorescence images were recorded using the *ChampChemi* Professional+ Image Station and analyzed using software Lane 1D v4.0. (*ChampChemi, Inc.*, China). All image-quantification were carried out using NIH ImageJ.^[1]^

■ **Experimental section**

SP microchip was prepared by latex dipping. 1.0 mM 1, 3, 3-trimethylindolinonaphthospirooxazine and thermoplastic polyurethane Tecoflex® (0.5 % PU in Ethanol) solution (200 nL) pipetted onto each pixel of the microchip. After the solvent dry, the SP microchip was performed to the multi-analytes testing (11 metal ion plus the pH = 5 control solution). On the microchip, AlCl_3_, CaCl_2_, CdCl_2_, CoCl_2_, CrCl_3_, CuCl_2_, FeCl_2_, HgCl_2_, MgCl_2_, NiCl_2_ and ZnCl_2_ (1 mM in water, 200 nL, pH = 5) and pH = 5 control aqueous solutions (HCl-NaCl) were spotted on corresponding pixels respectively.

The fluorescence responses of the SP microchip to different metal ions were recorded by fluorescence scanner in six channels (CH1: 450 nm, CH2: 480 nm, CH3: 505 nm, CH4: 535 nm, CH5: 570 nm and CH6: 605 nm, with 365 nm UV light excitation). The data processing consisted on the integration of the fluorescence intensity per pixel of the SP microchip before and after the solutions of metal ions spotted.

**■ Data analysis**

***Linear discriminant analysis (LDA)***

***Table S1-4.*** The jack-knifed classification procedure on integrated SP multi-states array and SP array in dark, UV or Vis stimulation state. Summary of 84 samples classification to 11 metal ions and 1 control analytes with cross-validation by LDA.

***Table S1***

| **Jackknifed Classification Matrix on SP Multi-States Array** | | | | | | | | | | | | | |
| --- | --- | --- | --- | --- | --- | --- | --- | --- | --- | --- | --- | --- | --- |
|  | Al^3+^ | Ca^2+^ | Cd^2+^ | Co^2+^ | Cr^3+^ | Cu^2+^ | Fe^2+^ | Hg^2+^ | Mg^2+^ | Ni^2+^ | Zn^2+^ | pH=5 | Correct |
| Al^3+^ | 7 | 0 | 0 | 0 | 0 | 0 | 0 | 0 | 0 | 0 | 0 | 0 | 100% |
| Ca^2+^ | 0 | 7 | 0 | 0 | 0 | 0 | 0 | 0 | 0 | 0 | 0 | 0 | 100% |
| Cd^2+^ | 0 | 0 | 7 | 0 | 0 | 0 | 0 | 0 | 0 | 0 | 0 | 0 | 100% |
| Co^2+^ | 0 | 0 | 0 | 7 | 0 | 0 | 0 | 0 | 0 | 0 | 0 | 0 | 100% |
| Cr^3+^ | 0 | 0 | 0 | 0 | 7 | 0 | 0 | 0 | 0 | 0 | 0 | 0 | 100% |
| Cu^2+^ | 0 | 0 | 0 | 0 | 0 | 7 | 0 | 0 | 0 | 0 | 0 | 0 | 100% |
| Fe^2+^ | 0 | 0 | 0 | 0 | 0 | 0 | 7 | 0 | 0 | 0 | 0 | 0 | 100% |
| Hg^2+^ | 0 | 0 | 0 | 0 | 0 | 0 | 0 | 7 | 0 | 0 | 0 | 0 | 100% |
| Mg^2+^ | 0 | 0 | 0 | 0 | 0 | 0 | 0 | 0 | 7 | 0 | 0 | 0 | 100% |
| Ni^2+^ | 0 | 0 | 0 | 0 | 0 | 0 | 0 | 0 | 0 | 7 | 0 | 0 | 100% |
| Zn^2+^ | 0 | 0 | 0 | 0 | 0 | 0 | 0 | 0 | 0 | 0 | 7 | 0 | 100% |
| pH=5 | 0 | 0 | 0 | 0 | 0 | 0 | 0 | 0 | 0 | 0 | 0 | 7 | 100% |
| Total | 7 | 7 | 7 | 7 | 7 | 7 | 7 | 7 | 7 | 7 | 7 | 7 | 100% |
| N = 84 | | | | | N Correct = 84 | | | | | Proportion Correct = 1.000 | | | |

***Table S***2

| **Jackknifed Classification Matrix on SP Array in Dark State** | | | | | | | | | | | | | |
| --- | --- | --- | --- | --- | --- | --- | --- | --- | --- | --- | --- | --- | --- |
|  | Al^3+^ | Ca^2+^ | Cd^2+^ | Co^2+^ | Cr^3+^ | Cu^2+^ | Fe^2+^ | Hg^2+^ | Mg^2+^ | Ni^2+^ | Zn^2+^ | pH=5 | Correct |
| Al^3+^ | 7 | 0 | 0 | 0 | 0 | 0 | 0 | 0 | 0 | 0 | 0 | 0 | 100% |
| Ca^2+^ | 0 | 3 | 3 | 0 | 0 | 0 | 0 | 0 | 0 | 0 | 0 | 1 | 43% |
| Cd^2+^ | 0 | 2 | 5 | 0 | 0 | 0 | 0 | 0 | 0 | 0 | 0 | 0 | 71% |
| Co^2+^ | 0 | 2 | 1 | 2 | 0 | 0 | 0 | 0 | 1 | 0 | 0 | 1 | 29% |
| Cr^3+^ | 0 | 0 | 0 | 0 | 7 | 0 | 0 | 0 | 0 | 0 | 0 | 0 | 100% |
| Cu^2+^ | 0 | 0 | 0 | 0 | 0 | 7 | 0 | 0 | 0 | 0 | 0 | 0 | 100% |
| Fe^2+^ | 0 | 0 | 0 | 0 | 0 | 0 | 7 | 0 | 0 | 0 | 0 | 0 | 100% |
| Hg^2+^ | 0 | 0 | 0 | 0 | 0 | 0 | 0 | 7 | 0 | 0 | 0 | 0 | 100% |
| Mg^2+^ | 0 | 0 | 0 | 0 | 0 | 0 | 0 | 0 | 7 | 0 | 0 | 0 | 100% |
| Ni^2+^ | 0 | 0 | 0 | 0 | 0 | 0 | 0 | 0 | 0 | 7 | 0 | 0 | 100% |
| Zn^2+^ | 0 | 0 | 0 | 0 | 0 | 0 | 0 | 0 | 0 | 0 | 7 | 0 | 100% |
| pH=5 | 0 | 0 | 0 | 3 | 0 | 0 | 0 | 0 | 0 | 0 | 0 | 4 | 57% |
| Total | 7 | 7 | 9 | 5 | 7 | 7 | 7 | 7 | 8 | 7 | 7 | 6 | 83% |
| N = 84 | | | | | N Correct = 70 | | | | | Proportion Correct = 0.833 | | | |

***Table S***3

| **Jackknifed Classification Matrix on SP Array in UV State** | | | | | | | | | | | | | |
| --- | --- | --- | --- | --- | --- | --- | --- | --- | --- | --- | --- | --- | --- |
|  | Al^3+^ | Ca^2+^ | Cd^2+^ | Co^2+^ | Cr^3+^ | Cu^2+^ | Fe^2+^ | Hg^2+^ | Mg^2+^ | Ni^2+^ | Zn^2+^ | pH=5 | Correct |
| Al^3+^ | 7 | 0 | 0 | 0 | 0 | 0 | 0 | 0 | 0 | 0 | 0 | 0 | 100% |
| Ca^2+^ | 0 | 5 | 0 | 0 | 0 | 0 | 0 | 0 | 0 | 0 | 0 | 2 | 71% |
| Cd^2+^ | 0 | 0 | 3 | 2 | 0 | 0 | 0 | 0 | 0 | 0 | 0 | 2 | 43% |
| Co^2+^ | 0 | 0 | 1 | 2 | 0 | 0 | 0 | 0 | 2 | 0 | 0 | 2 | 29% |
| Cr^3+^ | 0 | 0 | 0 | 0 | 7 | 0 | 0 | 0 | 0 | 0 | 0 | 0 | 100% |
| Cu^2+^ | 0 | 0 | 0 | 0 | 0 | 7 | 0 | 0 | 0 | 0 | 0 | 0 | 100% |
| Fe^2+^ | 0 | 0 | 0 | 0 | 0 | 0 | 7 | 0 | 0 | 0 | 0 | 0 | 100% |
| Hg^2+^ | 0 | 0 | 0 | 0 | 0 | 1 | 0 | 5 | 1 | 0 | 0 | 0 | 71% |
| Mg^2+^ | 0 | 0 | 0 | 0 | 0 | 0 | 0 | 0 | 7 | 0 | 0 | 0 | 100% |
| Ni^2+^ | 0 | 0 | 0 | 0 | 0 | 0 | 0 | 0 | 0 | 7 | 0 | 0 | 100% |
| Zn^2+^ | 0 | 0 | 0 | 0 | 0 | 0 | 0 | 0 | 0 | 0 | 7 | 0 | 100% |
| pH=5 | 0 | 0 | 0 | 1 | 0 | 0 | 0 | 0 | 0 | 0 | 0 | 6 | 86% |
| Total | 7 | 5 | 4 | 5 | 7 | 8 | 7 | 5 | 10 | 7 | 7 | 12 | 83% |
| N = 84 | | | | | N Correct = 70 | | | | | Proportion Correct = 0.833 | | | |

***Table S***4

| **Jackknifed Classification Matrix on SP Array in Vis State** | | | | | | | | | | | | | |
| --- | --- | --- | --- | --- | --- | --- | --- | --- | --- | --- | --- | --- | --- |
|  | Al^3+^ | Ca^2+^ | Cd^2+^ | Co^2+^ | Cr^3+^ | Cu^2+^ | Fe^2+^ | Hg^2+^ | Mg^2+^ | Ni^2+^ | Zn^2+^ | pH=5 | Correct |
| Al^3+^ | 7 | 0 | 0 | 0 | 0 | 0 | 0 | 0 | 0 | 0 | 0 | 0 | 100% |
| Ca^2+^ | 0 | 3 | 0 | 0 | 0 | 0 | 0 | 0 | 0 | 0 | 0 | 4 | 43% |
| Cd^2+^ | 0 | 0 | 7 | 0 | 0 | 0 | 0 | 0 | 0 | 0 | 0 | 0 | 100% |
| Co^2+^ | 0 | 0 | 0 | 7 | 0 | 0 | 0 | 0 | 0 | 0 | 0 | 0 | 100% |
| Cr^3+^ | 0 | 0 | 0 | 0 | 7 | 0 | 0 | 0 | 0 | 0 | 0 | 0 | 100% |
| Cu^2+^ | 0 | 0 | 0 | 0 | 0 | 7 | 0 | 0 | 0 | 0 | 0 | 0 | 100% |
| Fe^2+^ | 0 | 0 | 0 | 0 | 0 | 0 | 7 | 0 | 0 | 0 | 0 | 0 | 100% |
| Hg^2+^ | 0 | 0 | 0 | 0 | 0 | 0 | 0 | 7 | 0 | 0 | 0 | 0 | 100% |
| Mg^2+^ | 0 | 0 | 0 | 0 | 0 | 0 | 0 | 0 | 7 | 0 | 0 | 0 | 100% |
| Ni^2+^ | 0 | 1 | 0 | 0 | 0 | 0 | 0 | 0 | 0 | 6 | 0 | 0 | 86% |
| Zn^2+^ | 0 | 0 | 0 | 0 | 0 | 0 | 0 | 0 | 0 | 0 | 7 | 0 | 100% |
| pH=5 | 0 | 0 | 0 | 0 | 0 | 0 | 0 | 0 | 0 | 0 | 0 | 7 | 100% |
| Total | 7 | 4 | 7 | 7 | 7 | 7 | 7 | 7 | 7 | 6 | 7 | 11 | 94% |
| N = 84 | | | | | N Correct = 79 | | | | | Proportion Correct = 0.940 | | | |

***Table S5***. Graphic representation of LDA results on SP in dark, UV or Vis stimulation state. The figures in table display the distribution of the clusters in the same 3-dimention space, and the corresponding magnified images. From the LDA results table, we can find the near and confused spatial distribution of the clusters in each single SP states arrays, which reveals the small differential of fluorescent signal of the metal ions in these analysis systems.

| **LDA Result on SP Array in Dark State** |
| --- |
| 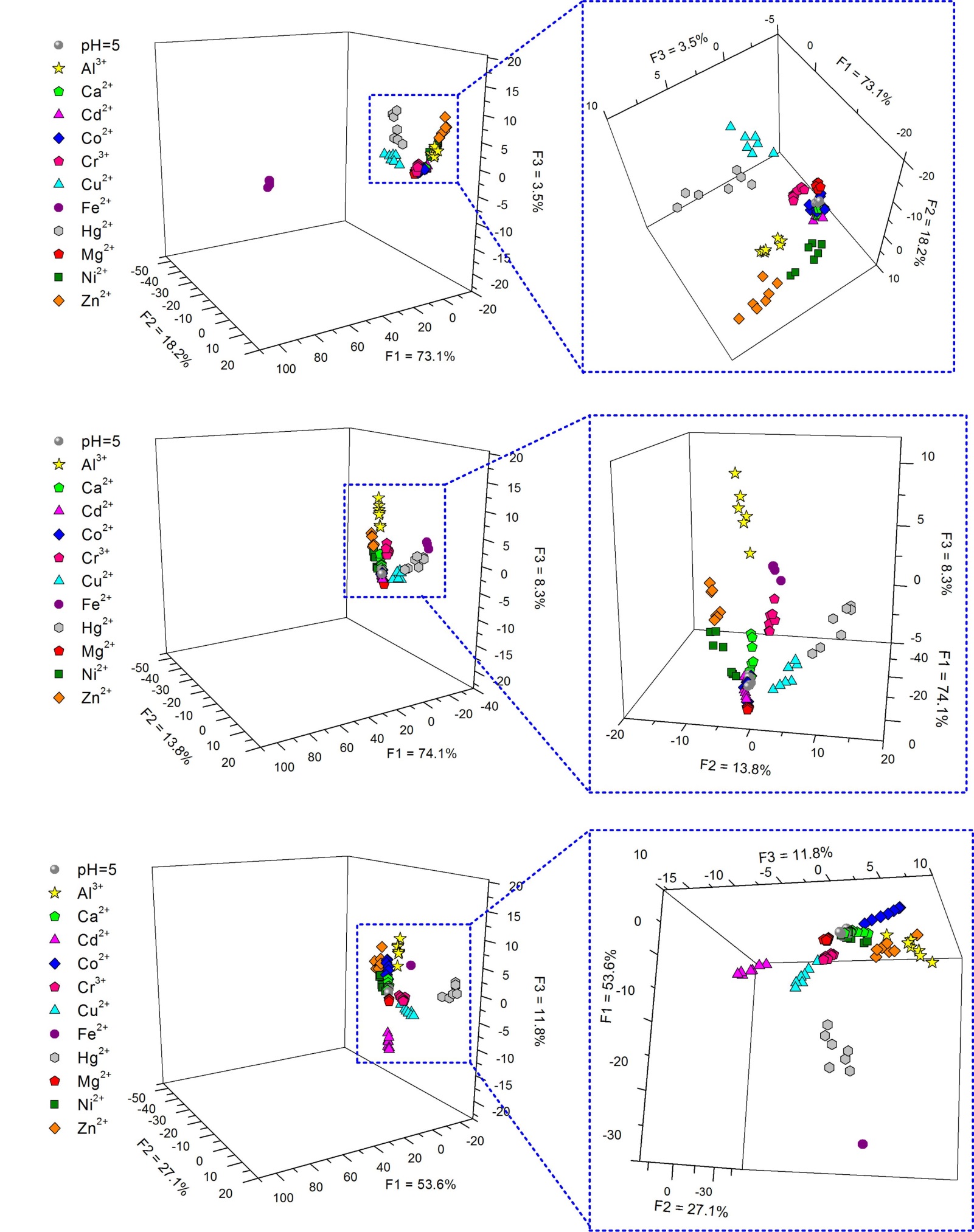 |
| **LDA Result on SP Array in UV State** |
| 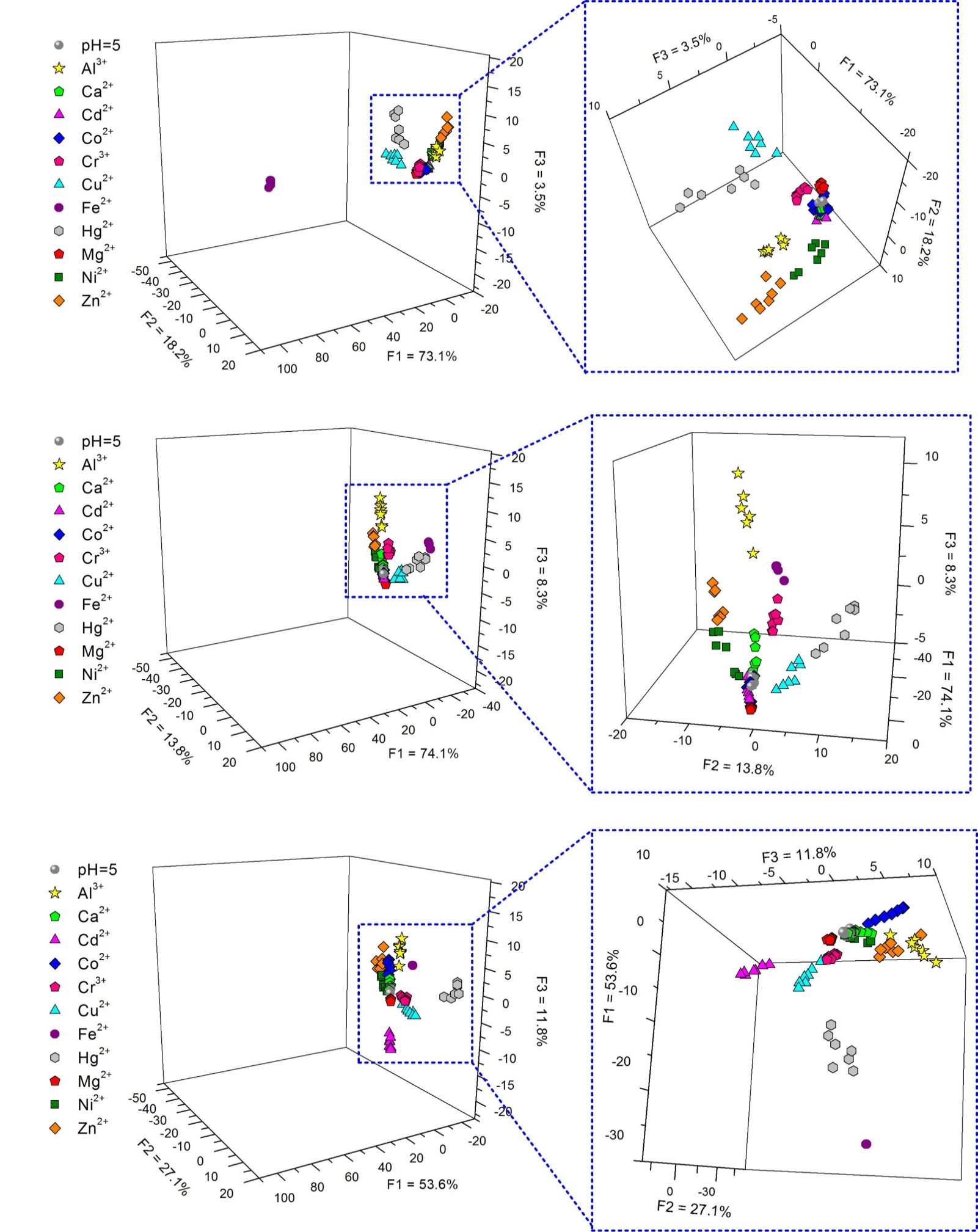 |
| **LDA Result on SP Array in Vis State** |
| 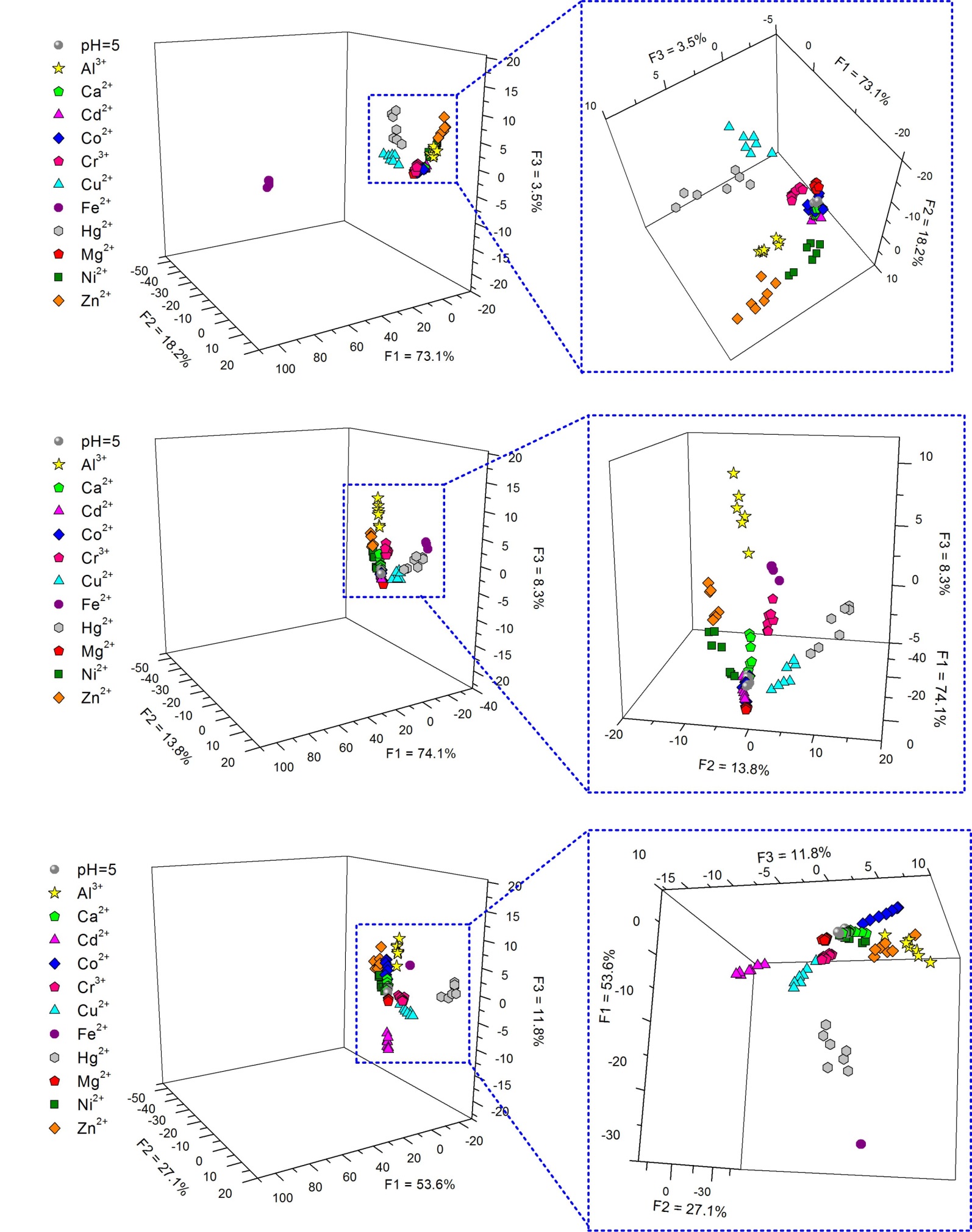 |

***Hierarchical clustering analysis (HCA)***

***Figure S1.*** Dendrogram of euclidean distance between 84 samples with ward linkage in SP multi-states array.


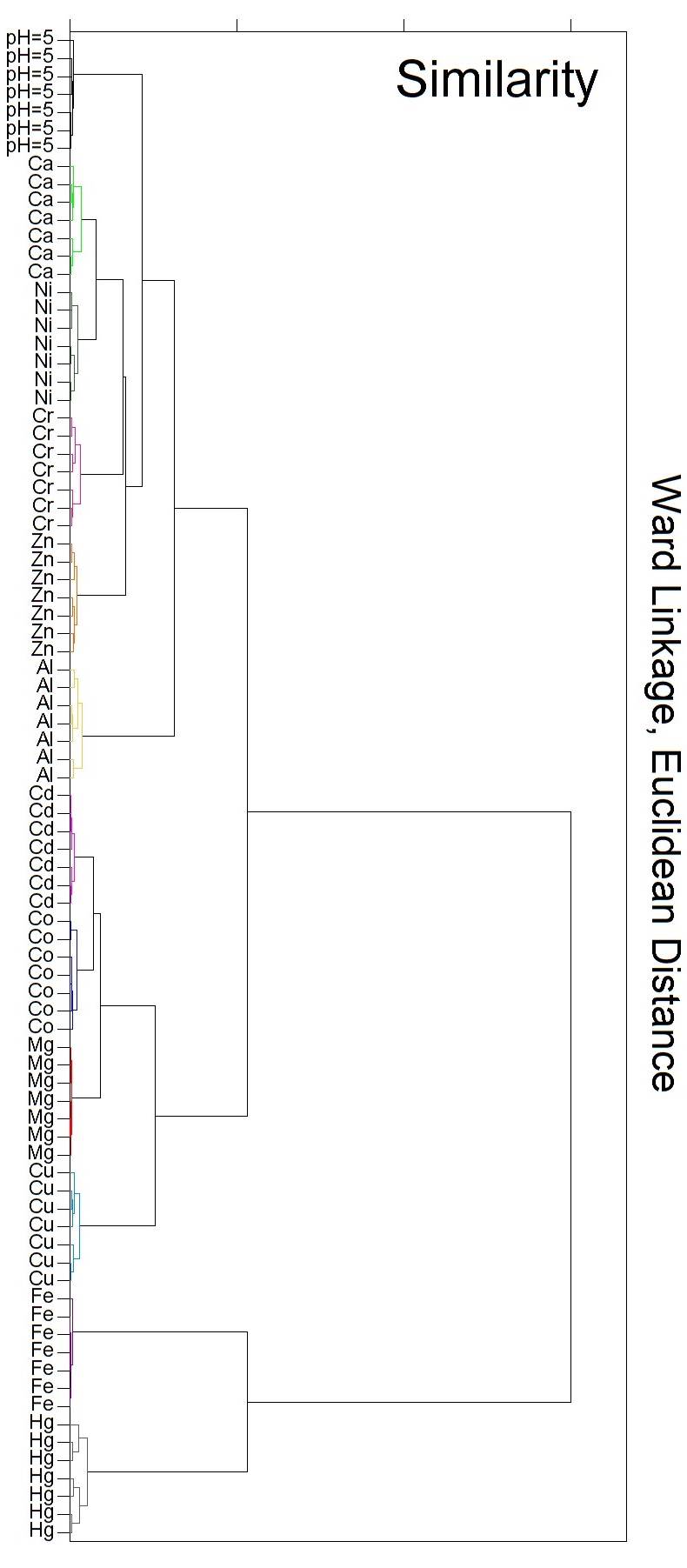


HCA is an unsupervised method of multivariate analysis, seeking classification of the samples by measuring the interpoint distances (in this study, the Euclidean distance) between all samples in the n-dimensional space resulting from n-numbers of studied features. In this work, we defined the cluster by Ward’s (minimum variance) method, which takes into consideration the minimum amount of variance between the samples and analytes to define a cluster.

***LDA analysis of 16 kinds of natural mineral water***

***Table S6.*** The jack-knifed classification procedure on SP multi-states array. Summary of 255 samples classification to 16 kinds of natural mineral water and 1 control analytes with cross-validation by LDA.


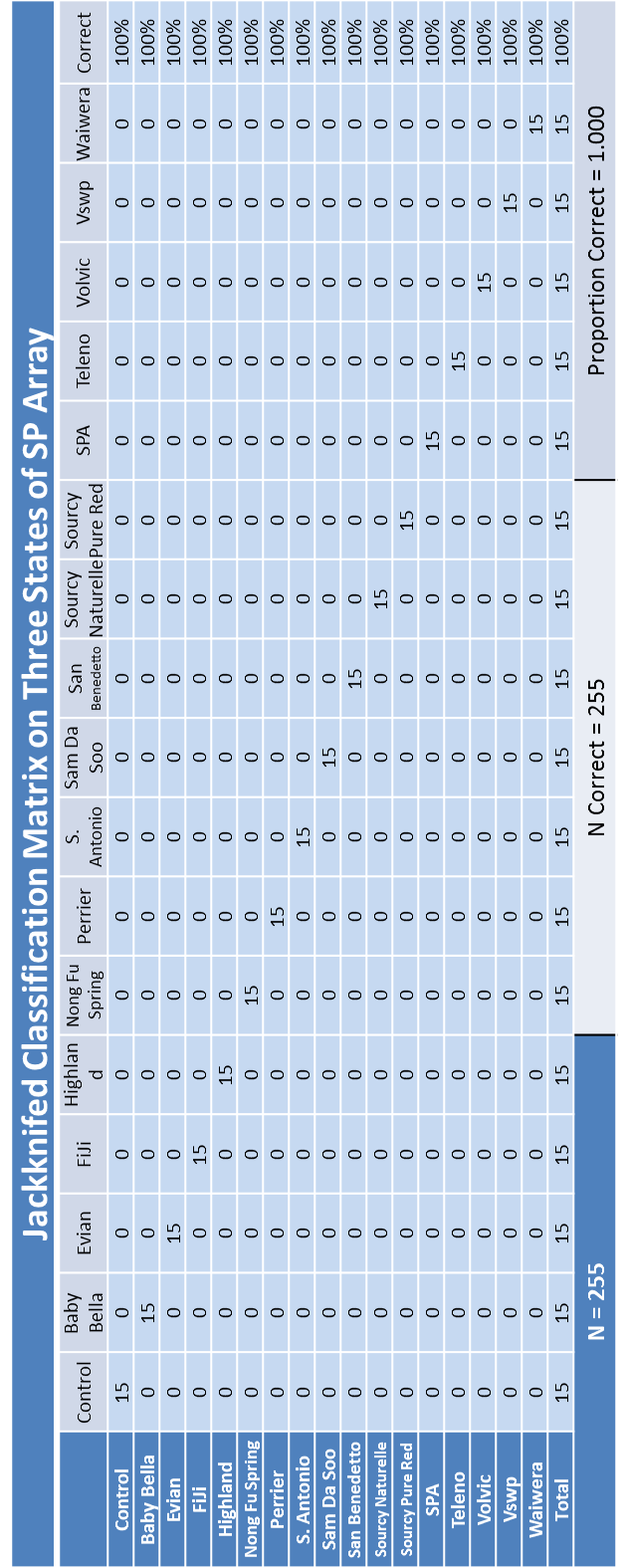


***LDA analysis of 11 metal ions in human serum***

***Figure S2.*** Discriminant analysis of 11 metal ions in human serum on SP multi-states array.


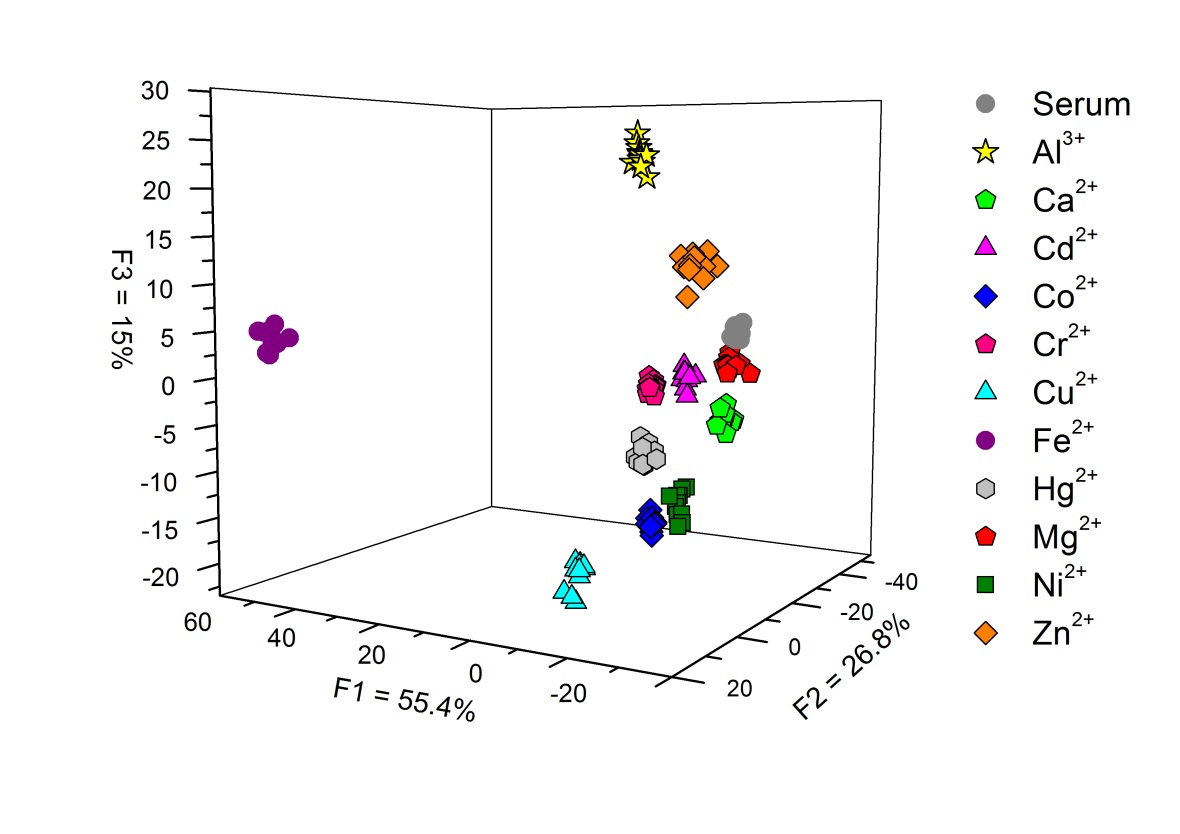


The graph of LDA result shows a clear clustering of the 11 metal ions analytes. The results demonstrate that the SP microchip can perform powerful identification of metal ions in complex physiological environment with low risk of matrix interference, suggesting its potential application in complex physiological environment.

***Table S7.*** The jack-knifed classification procedure on SP multi-states array. Summary of 132 samples classification to 11 metal ions in human serum and 1 control (serum) analytes with cross-validation by LDA.

| **Jackknifed Classification Matrix on SP Multi-States Array** | | | | | | | | | | | | | |
| --- | --- | --- | --- | --- | --- | --- | --- | --- | --- | --- | --- | --- | --- |
|  | Al^3+^ | Ca^2+^ | Cd^2+^ | Co^2+^ | Cr^3+^ | Cu^2+^ | Fe^2+^ | Hg^2+^ | Mg^2+^ | Ni^2+^ | Zn^2+^ | Serum | Correct |
| Al^3+^ | 11 | 0 | 0 | 0 | 0 | 0 | 0 | 0 | 0 | 0 | 0 | 0 | 100% |
| Ca^2+^ | 0 | 11 | 0 | 0 | 0 | 0 | 0 | 0 | 0 | 0 | 0 | 0 | 100% |
| Cd^2+^ | 0 | 0 | 11 | 0 | 0 | 0 | 0 | 0 | 0 | 0 | 0 | 0 | 100% |
| Co^2+^ | 0 | 0 | 0 | 11 | 0 | 0 | 0 | 0 | 0 | 0 | 0 | 0 | 100% |
| Cr^3+^ | 0 | 0 | 0 | 0 | 11 | 0 | 0 | 0 | 0 | 0 | 0 | 0 | 100% |
| Cu^2+^ | 0 | 0 | 0 | 0 | 0 | 11 | 0 | 0 | 0 | 0 | 0 | 0 | 100% |
| Fe^2+^ | 0 | 0 | 0 | 0 | 0 | 0 | 11 | 0 | 0 | 0 | 0 | 0 | 100% |
| Hg^2+^ | 0 | 0 | 0 | 0 | 0 | 0 | 0 | 11 | 0 | 0 | 0 | 0 | 100% |
| Mg^2+^ | 0 | 0 | 0 | 0 | 0 | 0 | 0 | 0 | 11 | 0 | 0 | 0 | 100% |
| Ni^2+^ | 0 | 0 | 0 | 0 | 0 | 0 | 0 | 0 | 0 | 11 | 0 | 0 | 100% |
| Zn^2+^ | 0 | 0 | 0 | 0 | 0 | 0 | 0 | 0 | 0 | 0 | 11 | 0 | 100% |
| Serum | 0 | 0 | 0 | 0 | 0 | 0 | 0 | 0 | 0 | 0 | 0 | 11 | 100% |
| Total | 11 | 11 | 11 | 11 | 11 | 11 | 11 | 11 | 11 | 11 | 11 | 11 | 100% |
| N = 132 | | | | | N Correct = 132 | | | | | Proportion Correct = 1.000 | | | |

**■ Mathematical deduction**

LDA is a classical statistical approach for supervised dimensionality reduction. LDA is also a special case of discriminant analyses where the discriminant rule is based on linear combinations of features (e.g., sensors responses) that best separate two or more analytes. Using the defined group classes, LDA aims to maximize the ratio of the between-the-class distance to the within-the-class distance, thus maximizing the class discrimination. As in PCA, the linear combinations of the features are found by solving an eigenvalue problem. The weights of the linear discriminant functions are determined from the eigenvector of the matrix D.

*D* = *G*^-1^ *H w* = *λw*

where *λ* is the eigenvalue

The matrix *G* is found from the covariance matrix *C* of the different groups *g* following:

*G* = (*n*
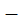
*g*) *C* = (*n*
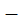
*g*)
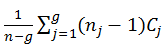


and
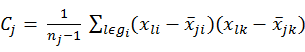


for *n* equal to the total number of observations, *n_j_* equal to number of observationin the group *j* and *l* is one observation of the *j*th group *g_j_.*

The matrix *H* explains the distribution of the group means *g_j_* over the total average
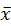
,

H =
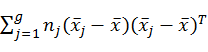


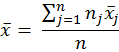


Solving the eigenvalue problem *D* = *G*^-1^ *H w* = *λw* will derive a list of eigenvalues (*λ*) and eigenvectors (*w*). The eigenvector *w_1_* associated with the greatest eigenvalue *λ_1_* provides the first discriminant function *s_1_*:


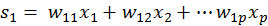


Utilizing the rest of the x-data the following eigenvalue *λ_2_* is calculated along with eigenvector *w_2_*, to obtain the second discriminant function, *s_2_*:


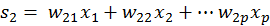


The calculation of the discriminant functions continues until all are determined. In order to classify any observation, the vector response (in case of sensor arrays) is evaluated in the discriminant functions in order to transform the vector of the raw data in the coordinates within the discriminant space. The observation is then assigned to the group to which it has the minimal Euclidean distance:


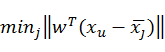
 with *j* = 1, 2, …*g*

The results of the classification could be represented in a confusion matrix, which is a matrix that contains the numbers of correctly classified objects in each class on the main diagonal and the misclassified objects in the off-diagonal. The confusion matrix often over estimates the accuracy of the classification due to the bias imposed when a sample classification is attempted while using a training-set that contains the same observation. The cross-validation (leave-one-out) routine is used to test the predictability of the sensor array by leaving one observation out of the set at the time, and it uses the rest of the data as a training set to generate the linear discriminant function, which is then used to place the excluded observation (data point) within the correct cluster. This is performed for each observation, and the overall ability to classify the observations describes the quality and predictability of the array. As in the case of the confusion matrix, the cross-validated results can be represented in a jackknifed matrix.

First, we assume our conditions:

(a) $\begin{matrix} x_{ij} & i.i.d\sim u_{i}+\sigma\varepsilon_{ij} \end{matrix}$

Where $x_{ij}$ is an observation of the ith group and {$\varepsilon_{ij}$} is a strong white noise with density function f. (i.e. $\varepsilon_{ij}$i.i.d with mean zero and identical variance)

(b) $\Delta:=\min_{i\neq j} |u_{i}-u_{j}|$ satisfied $\Delta>>\sigma$

($\Delta$is the distinction of our classification and $\varepsilon_{ij}$ is the uncertainty of our measurements, condition (b) guarantee that our method works )

By the well-known Kolmogorov's Law of the Iterated Logarithm,[[^2^](#_ENREF_1)] with the increase of $n_{i}$, we get a more accurate estimator $\bar{x}_{i}$, thus

$$\lim\sup_{n_{i}} \frac{|\bar{x}_{i}-u_{i}|n_{i}}{\sqrt{2n_{i}\mathrm{loglog}n_{i}}}=1$$

so we have

$$|\bar{x}_{i}-u_{i}|\leq\frac{\sqrt{2n_{i}\mathrm{loglog}n_{i}}}{n_{i}}$$

observing that

{$x_{ij}$is classified successfully} $\begin{matrix} \Leftrightarrow& \{|x_{ij}-\bar{x}_{i}|\leq\min_{k\neq i} |x_{ij}-\bar{x}_{k}|\} & \supset\end{matrix}$

$$\{|x_{ij}-u_{i}|\leq\frac{\Delta}{2}-2\max_{i} \frac{\sqrt{2\mathrm{loglog}n_{i}}}{n_{i}}\}$$

so,

P{$x_{ij}$is classified successfully }$\geq\int_{-(\frac{\Delta}{2\sigma}-\frac{2}{\sigma}\max_{i} \frac{\sqrt{2\mathrm{loglog}n_{i}}}{n_{i}})}^{\frac{\Delta}{2\sigma}-\frac{2}{\sigma}\max_{i} \frac{\sqrt{2\mathrm{loglog}n_{i}}}{n_{i}}} f(t)dt$

**■ References:**

1. W. S. Rasband, Image J, U. S. *National Institutes of Health, Bethesda, Maryland, USA,* <http://rsb.info.nih.gov/ij/>, (1997-2008).
2. A. Kolmogoroff, Über das Gesetz des iterierten Logarithmus. *Mathematische Annalen* **1929**, *101*, 126-135.
